# Sirt7 inhibits Sirt1-mediated activation of Suv39h1

**DOI:** 10.1080/15384101.2018.1486166

**Published:** 2018-07-25

**Authors:** Poonam Kumari, Daniela Popescu, Shijing Yue, Eva Bober, Alessandro Ianni, Thomas Braun

**Affiliations:** a Department of Cardiac Development and Remodeling, Max-Planck-Institute for Heart and Lung Research, Bad Nauheim, Germany; b The State Key Laboratory of Medicinal Chemical Biology, School of Medicine, Nankai University, Tianjin, China; c The State International Science & Technology Cooperation Base of Tumor Immunology and Biological Vaccines, Nankai University, Tianjin, China

**Keywords:** Sirtuins, Sirt1, Sirt7, acetylation, Suv39h1, histones, methylation

## Abstract

Sirtuins regulate a variety of cellular processes through protein deacetylation. The best-known member of mammalian sirtuin family, Sirt1, plays important roles in the maintenance of cellular homeostasis by regulating cell metabolism, differentiation and stress responses, among others. Sirt1 activity requires tight regulation to meet specific cellular requirements, which is achieved at different levels and by specific mechanisms. Recently, a regulatory loop between Sirt1 and another sirtuin, Sirt7, was identified. Sirt7 inhibits Sirt1 autodeacetylation at K230 and activation thereby preventing Sirt1-mediated repression of adipocyte differentiation by inhibition of the PPARγ gene. Here, we extend the regulatory complexity of Sirt7-dependent restriction of Sirt1 activity by demonstrating that Sirt7 reduces activation of a previously described prominent Sirt1 target, the histone methyltransferase Suv39h1. We show that removal of the acetyl-group at K230 in Sirt1 due to the absence of Sirt7 leads to hyperactivation of Sirt1 and thereby to constantly increased activity of Suv39h1.

## Introduction

Sirtuins are highly conserved protein deacetylases that perform crucial regulatory tasks to maintain cellular homeostasis. In mammals, seven sirtuins (Sirt1 – Sirt7) have been identified which are involved -among other processes- in the regulation of cellular metabolism, proliferation, differentiation, and responses to stress signals [1,2]. Regulation of such different biological functions is mediated by deacetylation of critical cellular targets such as p53, FOXO and other transcription factors and enzymes as well as by the control of chromatin dynamics in response to internal or external stimuli. Sirtuins deacetylate histones H1, H3, H4 at different lysine residues thus contributing to gene silencing and heterochromatin formation [2–5]. Sirtuins also control activation of histone modifiers such as methyltransferases and acetyltransferases, thereby regulating a complex network promoting chromatin compaction [3].

Sirt1 exerts many effects that are beneficial for cellular survival such as improving metabolism, strengthening stress resistance and securing genome integrity [2,6–8]. In general, Sirt1 is believed to promote organismal health and delay aging. Inactivation of Sirt1 results either in embryonic/early postnatal lethality or – in the small percentage of surviving individuals – in premature aging. Correspondingly, increased Sirt1 expression promotes healthy aging [9–13]. However, Sirt1 overexpression and/or increased activity seem to exert beneficial effects when restrained to a relatively narrow range. Only moderate Sirt1 overexpression prevents heart pathologies, whereas high-level (12-times the endogenous expression level) expression is detrimental for cardiac physiology [14,15]. Thus, tight regulation of Sirt1 activity is a prerequisite to maintain cellular homeostasis. Sirt1 expression and activity are regulated at the transcription level, via mRNA stability and by several posttranslational modifications of the Sirt1 protein [16–18]. Recently, activation of Sirt1 by autodeacetylation at the lysine residue K230 was also documented [19], which is negatively regulated by another member of the sirtuin family, Sirt7. Absence of Sirt7 leads to hyperactive, deacetylated Sirt1 causing lipodystrophy in Sirt7 knockout mice due to repression of the PPARγ gene [19].

Here, we describe another consequence of Sirt1 inhibition by Sirt7. We demonstrate that the presence of hyperactive Sirt1 in Sirt7 deficient cells leads to permanent activation of the histone methyltransferase Suv39h1, a known target of Sirt1 [20], which subsequently results in higher histone 3 lysine 9 trimethylation (H3K9me3) levels within the repetitive pericentromeric heterochromatin.

## Results

### Sirt7 forms a molecular complex with the histone methyltransferase Suv39h1

**Figure 1.**
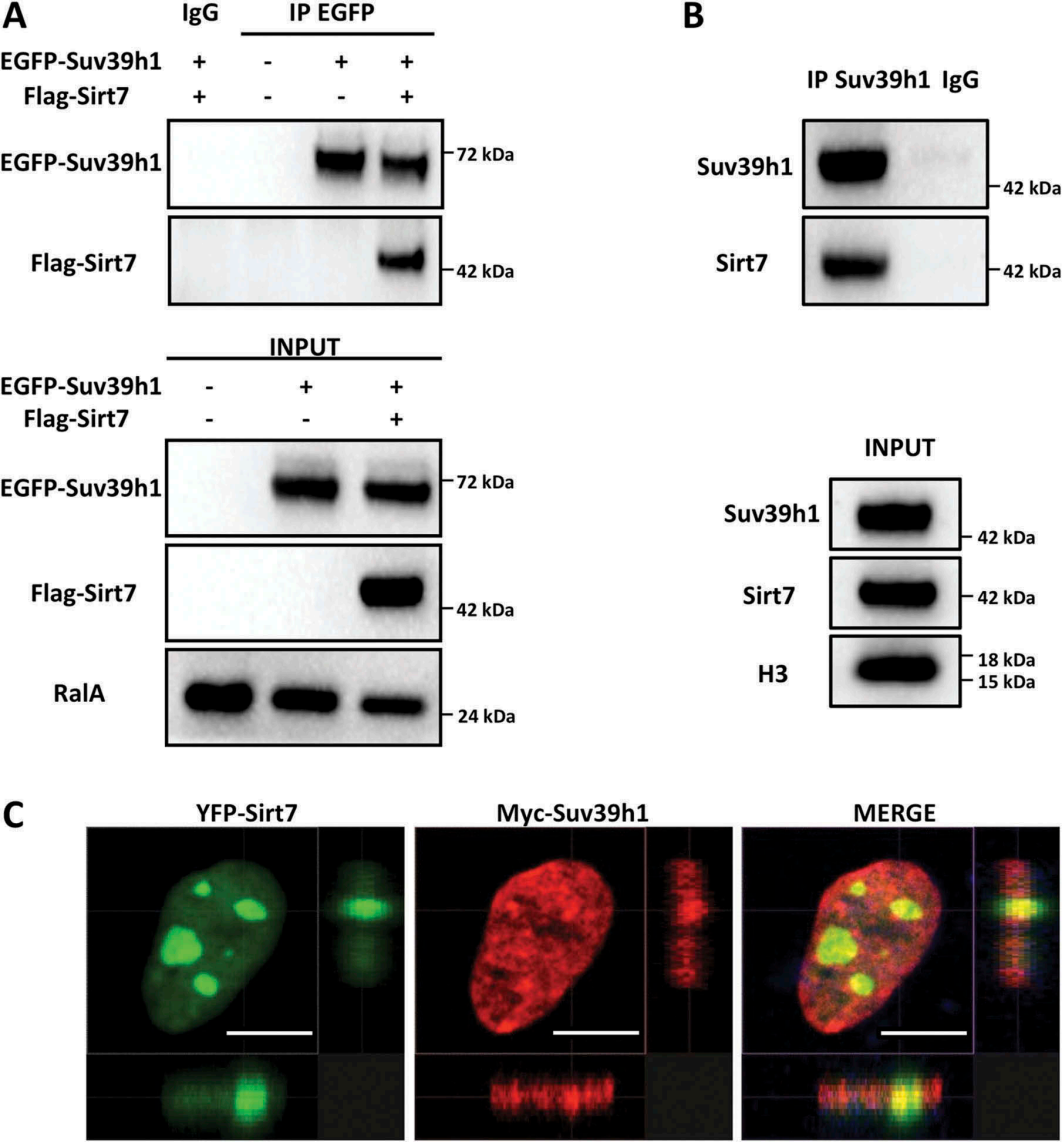
Sirt7 forms a complex with Suv39h1. (A) HEK293T cells were transfected with EGFP-Suv39h1 and Flag-Sirt7 constructs, harvested 48 h post-transfection and subjected to immunoprecipitation using an anti-EGFP antibody or a non-immune IgG as negative control, as indicated. The experiments were performed in presence of benzonase (0.15 U/µL) to degrade nucleic acids (upper panel). The inputs are shown in the lower panel. A representative picture of 3 independent experiments is shown. Antibodies used for WB analysis are indicated on the left. (B) Primary MEFs were immunoprecipitated as in (A) using anti-Suv39h1 antibody or IgG as a negative control. The immune-complexes were resolved by western blot and reacted with anti-Sirt7 and anti-Suv39H1 antibodies as indicated (upper panel). The inputs are shown in the lower panel. A representative image of 3 independent experiments is shown. (C) Co-localization of Myc-Suv39h1 and YFP-Sirt7 in U2OS cells. U2OS cells were subjected to immunofluorescence staining 48 h post-transfection. Pictures were captured with a Sp8 confocal microscope (Leica). Scale bar 10µm.

We have previously reported that Sirt7 interacts with and inhibits the Sirt1-mediated repression of PPARγ gene expression in adipocytes [19]. In contrast, Sirt7 promotes Sirt1 binding to rDNA together with DNA methyltransferase 1 (DNMT1) for induction of heterochromatin formation [21]. To further elucidate the role of Sirt7 on Sirt1, we wanted to explore whether Sirt7 also controls other Sirt1 effects on heterochromatin. Interestingly, in an overexpression system, we observed that Sirt7 co-immunoprecipitates with the histone methyltransferase Suv39h1 (Figure 1(A)), which is bound and activated by Sirt1 [20,22] suggesting formation of a tripartite Sirt7/Sirt1/Suv39h1 complex. Co-immunoprecipitation experiments in primary mouse embryonic fibroblasts (MEFs) confirmed that endogenous Sirt7 and Suv39h1 form a molecular complex (Figure 1(B)). Co-expression of Sirt7 and Suv39h1 in U2OS cells followed by immunofluorescence staining confirmed that Sirt7 and Suv39h1 co-localize in defined subcellular regions (Figure 1(C)).

### The activity of Suv39h1 is increased in Sirt7 deficient cells

**Figure 2.**
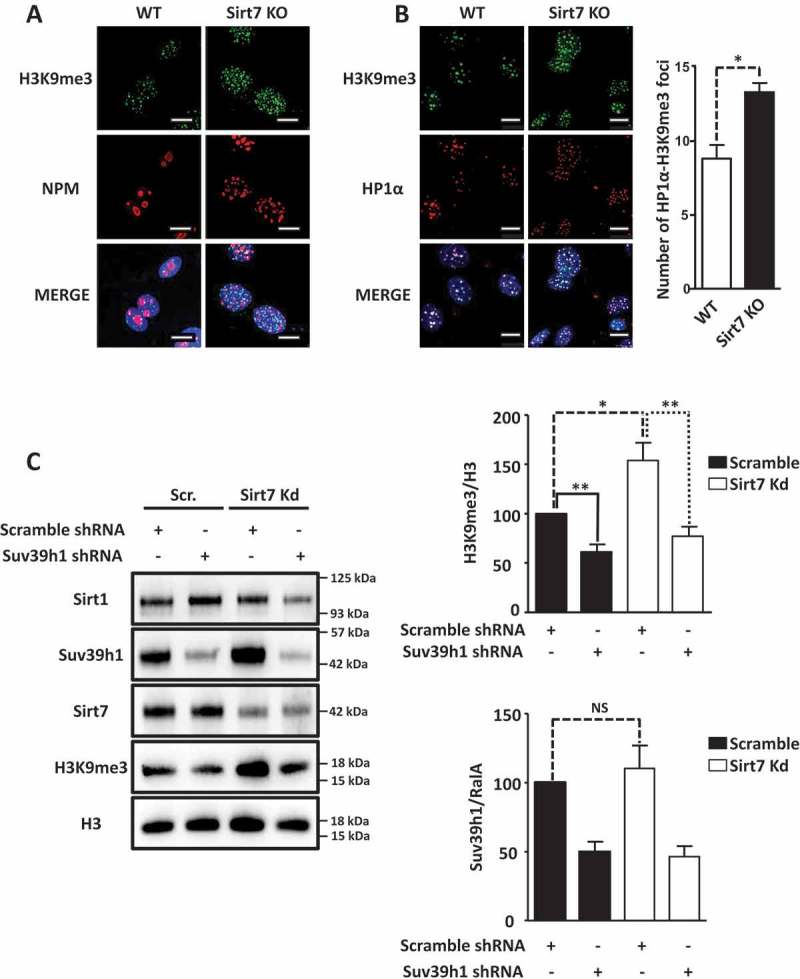
Inactivation of Sirt7 increases Suv39h1 activity. (A, B) Increased levels of H3K9me3 (A) and HP1α (B) in Sirt7 knockout MEFs (Sirt7 KO) visualized by immunofluorescence. Nucleoli were visualized by nucleophosmin (NPM) staining (A). Scale bars 10µm. Quantifications are shown in the panel on the right. (C) Western blot analysis of increased H3K9me3 levels indicates increased Suv39h1 activity after stable knockdown of Sirt7 in U2OS cells. Quantifications of H3K9me3 (upper panel) and Suv39h1 (lower panel) levels are shown on the right. Note that the increased H3K9me3 level in Sirt7 knockdown cells is reverted by knockdown of Suv39h1. The graph represents the average ± standard deviation of 4 independent experiments using four different, independently derived stable cell lines. *p < 0.05, **p < 0.01, NS: not significant.

To understand the consequences of increased Sirt1 activity due to the absence of Sirt7, we first determined Suv39h1 activity in Sirt7 knockout cells by immunofluorescence staining of the Suv39h1 substrate H3K9me3. H3K9me3 levels were clearly increased in Sirt7 knockout MEFs (Figure 2(A)) together with a higher number of HP1 alpha foci (HP1α), a known component of constitutive heterochromatin (Figure 2(B)). Notably, Sirt7 KO cells showed a disrupted nucleolar structure as visualized by staining for the nucleolar marker nucleophosmin (NPM), consistent with previous reports (Figure 2(A) [21];). To investigate whether the higher level of H3K9me3 observed in Sirt7 knockout cells is indeed due to increased activity of Suv39h1, we performed Suv39h1 and Sirt7 knock down experiments and western blot analyzes in U2OS cells. As expected, we observed that shRNA-mediated depletion of Suv39h1 decreased H3K9 trimethylation (Figure 2(C)). In contrast, introduction of Sirt7 shRNA led to a significant increase of H3K9me3 levels (Figure 2(C)), which was reverted by expression of shRNA against Suv39h1 in Sirt7 knock down cells (Figure 2(C)), thus demonstrating that a higher Suv39h1 activity is responsible for elevated H3K9me3 levels in Sirt7 deficient cells. The fact, that Suv39h1 expression level was not significantly affected by knockdown of Sirt7 (see the quantification in Figure 2(C)) additionally supports the presence of higher Suv39h1 catalytical activity in Sirt7 depleted cells.

### Sirt1 deacetylation and hyperactivation enhance Suv39h1 enzymatic activity in Sirt7 deficient cells

**Figure 3.**
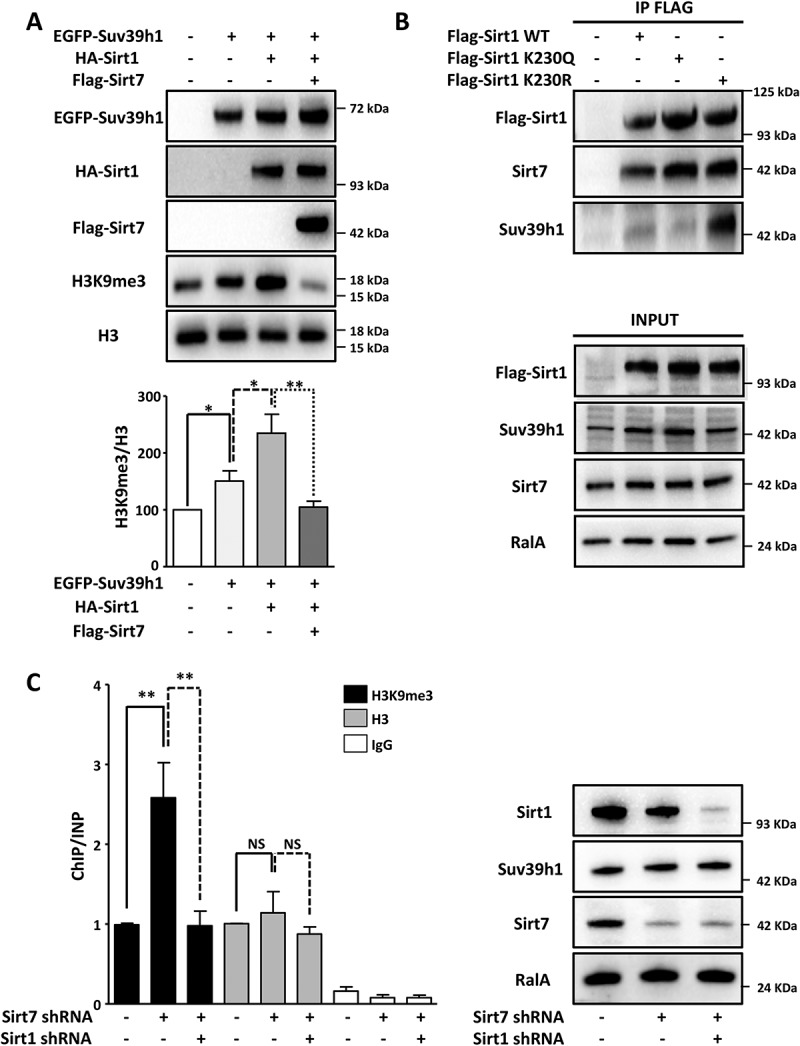
Hyperactive Sirt1 stimulates Suv39h1 activity. (A) Western blot analysis showing that overexpression of Sirt7 in HEK293T cells prevents Sirt1-mediated activation of Suv39h1. 48 h after transfection, cells were harvested and subjected to western blot analysis using the indicated antibodies. A representative picture of three independent experiments is shown. The histogram represents the quantification of H3K9me3 levels normalized to global H3 levels (H3K9me3/H3). Results were obtained from three independent biological replicates. *p < 0.05, **p < 0.01. (B) Western blot analysis showing that hyperactive Flag-Sirt1 K230R mutant binds endogenous Suv39h1 with high affinity while the Flag-Sirt1 K230Q mutant binds only weakly. Flag-tagged proteins were isolated from the HEK293T cells 48 h after transfection and analyzed by western blot with the indicated antibodies (upper panel). Inputs are shown in the lower panel. A representative picture of three independent experiments is shown. (C) ChIP analysis of H3K9me3 and total histone 3 (H3) at Sat2 elements indicates increased Suv39h1 activity after stable knockdown of Sirt7, which is then counteracted by the additional knockdown of Sirt1 in U2OS cell lines (left side). No significant changes in global H3 levels are apparent. The graph represents the average of 6 independent ChIP experiments for H3K9me3 and 3 independent experiments for total H3. For each ChIP experiment the qPCR reaction was performed in triplicate. **p < 0.01, NS: not significant. Western blot analysis demonstrated efficient knockdown of Sirt1 and Sirt7 (right side). No significant difference in Suv39h1 expression was observed between the scramble and stable knockdown cells.

Since Sirt1 positively regulates Suv39h1 activity [20] and Sirt1 becomes autodeacetylated at K230 and hyperactive in Sirt7 deficient cells [19], we speculated that hyperactivation of Sirt1 plays the decisive role for increased Suv39h1 activity in Sirt7 depleted cells. To corroborate this assumption, we expressed different combination of Sirt1, Sirt7 and Suv39h1 in HEK293T cells and monitored H3K9me3 levels. We found that Sirt1 together with Suv39h1 increased Suv39h1-dependent H3K9 methylation while addition of Sirt7 prevented this increase (Figure 3(A)). Since we previously attributed Sirt1 hyperactivity in Sirt7-deficient adipocytes to Sirt1 K230 autodeacetylation [19], we assumed that deacetylated Sirt1 might acquire a higher affinity to Suv39h1 and thereby more efficient Suv39h1 activation. Transfection of Sirt1 acetylation (Sirt1 K230Q) and deacetylation (K230R) mimicking mutants, and wildtype Sirt1 in HEK293T cells validated this assumption. We observed by co-immunoprecipitation that the hyperactive K230R deacetylation mimicking Sirt1 mutant interacted much more strongly with Suv39h1 compared to the Sirt1 K230Q mutant, which mimicks acetylation (Figure 3(B)).

### Inactivation of Sirt1 in Sirt7 depleted cells restores normal levels of H3K9me3

**Figure 4.**
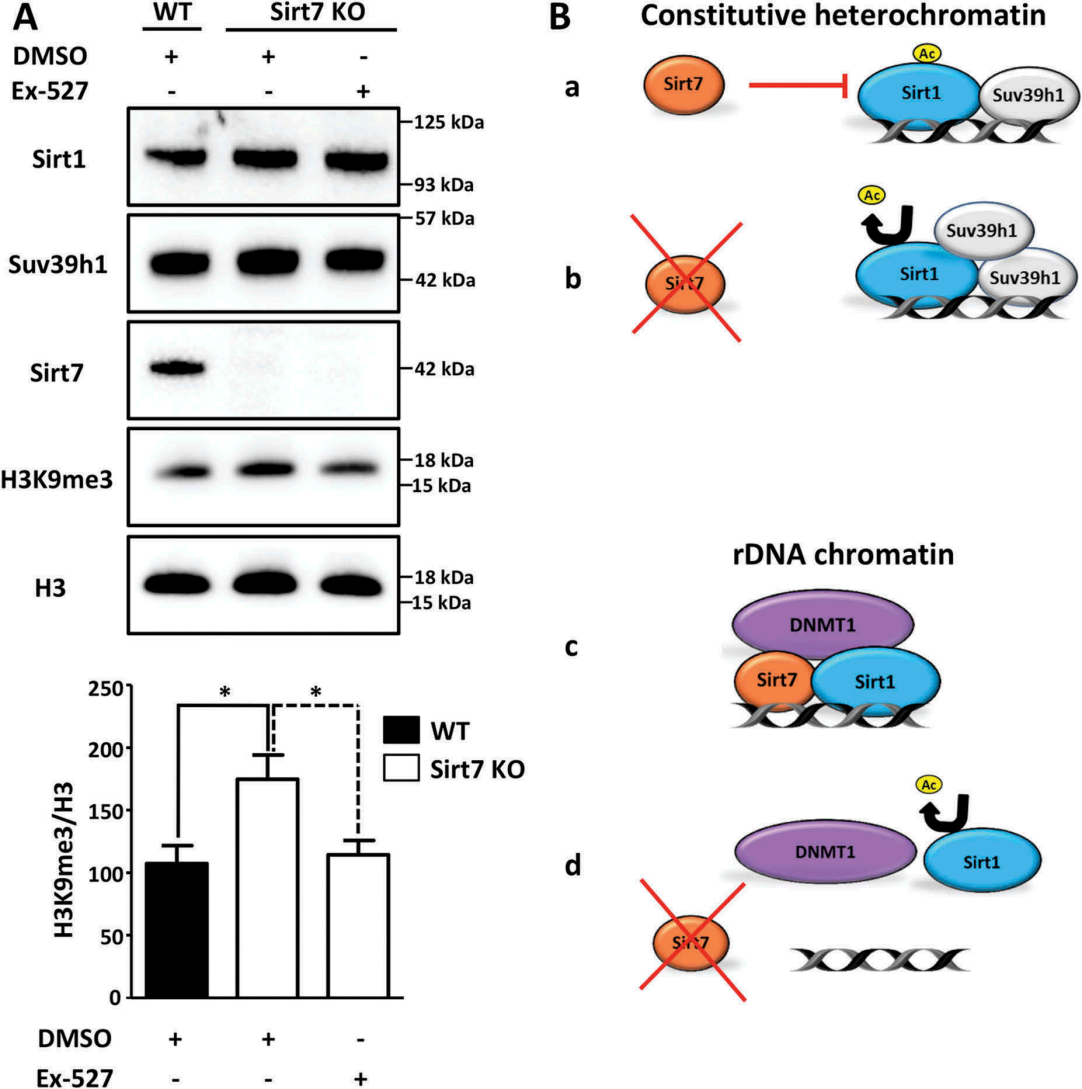
Inactivation of Sirt1 in Sirt7 knockout cells abolishes hypermethylation of H3K9. (A) Western blot analysis showing levels of Suv39h1 and H3K9me3 in wildtype (WT) and Sirt7 knockout MEFs (Sirt7 KO) untreated or treated with 10nM of Sirt1 specific inhibitor Ex-527 for 24 h. The quantification of H3K9me3 in relation to global H3 levels is presented in a histogram below. n = 3; *p < 0.05. (B) Schemes depicting the putative mechanisms of Sirt7-mediated regulation of Sirt1. See text for explanation.

Our results suggested a repression of Sirt1 activity by Sirt7 to maintain normal levels of H3K9 methylation within the pericentric chromatin. To directly demonstrate the impact of Sirt1-Sirt7 interactions on heterochromatin, we inactivated Sirt1 in Sirt7 depleted cells and analyzed the extent of H3K9 methylation in satellite DNA (Sat2; which is a part of the pericentric chromatin) by chromatin immunoprecipitation (ChIP; Figure 3(C)). We found that H3K9me3 level strongly increased upon Sirt7 depletion while Sirt1 inactivation prevented this effect (Figure 3(C)). Notably, we did not observe significant changes in the global levels of histone 3 (H3, Figure 3(C)) excluding the possibility that the higher levels of H3K9me3 at the Sat2 elements observed in Sirt7 deficient cells were a consequence of an altered content of H3. Additional experiments in Sirt7 knockout MEF cells yielded very similar results. The inhibition of Sirt1 with Ex-527, which specifically inhibits Sirt1 activity at nanomolar concentrations without affecting other sirtuins [23], restored normal H3K9me3 levels in Sirt7 KO cells compared to DMSO treated cells (Figure 4(A)).

## Discussion

Protein modification by acetylation/deacetylation is – similarly to phosphorylation/dephosphorylation – a frequently used mechanism to regulate functions of proteins [24]. Numerous examples of autocatalytic activation have been described for kinases and phosphatases, but only very few cases are known in which acetyltransferases or protein deacetylases undergo activation by autoacetylation or autodeacetylation [25–28]. Recently, we defined an autodeacetylation activity for Sirt1, which is inhibited by Sirt7. Sirt1 acquires higher catalytic activity to deacetylate different protein targets when autodeacetylated at lysine residue K230. Most importantly, using acetylation and deacetylation mimicking Sirt1 mutants, we demonstrated that Sirt1 autodeacetylation increases binding to Suv39h1 thus enabling more efficient Suv39h1 activation. Hyperactive Sirt1 very efficiently inhibits adipocyte differentiation by supressing the PPARγ gene [19], which explains why Sirt7 knockout mice are characterized by reduction of white adipose tissue depots [19]. We became interested to know whether disruption of the crosstalk between Sirt1 and Sirt7 has additional consequences. Here, we demonstrated that Sirt7 inhibits Sirt1 autodeacetylation to restrict Sirt1-dependent activation of Suv39h1 thereby preventing abnormally elevated Suv39h1-dependent H3K9 trimethylation levels (4(Ba)). Sirt7-mediated repression of Sirt1 and thereby of Suv39h1-dependent H3K9 trimethylation levels offers an opportunity to restrain excessive heterochromatin formation in metabolic conditions (i.e. increased NAD^+^ concentrations) favouring increased Suv39h1 activity. In the absence of Sirt7, Sirt1 is constantly deacetylated and causes increased Suv39h1 activity at constitutive pericentric heterochromatin resulting in high levels of H3K9me3 and disturbed heterochromatin structure (4(Bb)). Interestingly, the lack of Sirt7 seems to have different effects on rDNA repeats, which contain facultative heterochromatin [21]. At rDNA repeats the absence of Sirt7 leads to diminished DNMT1 and Sirt1 recruitment causing chromatin relaxation and rDNA instability (Figure 4(Bc), 4(Bd)). We reason that such differences might be explained by the inability of Sirt1 to get recruited to rDNA repeats in the absence of Sirt7 even when catalytically hyperactive Sirt1 is present (Figure 4(Bc), 4(Bd)) [21]. At present the significance of such opposite regulation of different chromatin regions remains elusive but is obviously biologically important, as illustrated by alterations of both constitutive and facultative nucleolar rDNA heterochromatin in Sirt7 knockout cells.

The nuclear sirtuin Sirt6 was recently shown to promote pericentric heterochromatin condensation and silencing of pericentric satellite repeat elements by deacetylation of lysine 18 of H3 (H3K18 [5];). In contrast, Sirt7 did not affect expression of satellite repeats despite the fact that Sirt7 is able to specifically deacetylate H3K18 [4,5]. The lack of Sirt7 influence on satellite repeats transcription is puzzling in view of the higher Suv39h1 recruitment to the pericentric heterochromatin demonstrated in our paper and requires further investigation. Although there is at present no specific hint that Sirt7 regulates Sirt6 activity directly, both deacetylases share several potential targets implying functional links [29]. Hence, it will be interesting to study the interactive network constituted by Sirt1, Sirt6 and Sirt7, which is based on direct interactions and shared targets, on the structure and compaction of the pericentric heterochromatin. Further experiments are needed to delineate the exact mechanisms and effects of each sirtuin on the pericentric chromatin.

Aberrant pericentric heterochromatin organization has been recognized to contribute to genomic instability, ageing and cancer progression [30–33]. It will be important to further investigate how the disrupted heterochromatin in Sirt7 deficient cells influences these processes. For instance, it has been proposed that loss of pericentromeric heterochromatin in cancer promotes chromosome instability [32]. Sirt7 is upregulated in different types of human cancers where it correlates with a more aggressive phenotype and poor prognosis [34]. We might assume that elevated Sirt7 levels inhibit Sirt1-mediated heterochromatin formation at pericentric regions thereby contributing to global genomic instability in cancer cells. On the other hand, more compact constitutive heterochromatin, observed in Sirt7 deficient cells, might prevent opening of chromatin regions that are in need for DNA repair causing accumulation of DNA damage and accelerated aging. In fact, recent evidence points to the importance of temporary chromatin relaxation even within highly compacted constitutive heterochromatin to allow DNA repair during stress responses [35]. The clear increase of constitutive heterochromatin in Sirt7 mutant cells seems to be in contrast to increased relaxation of facultative rDNA heterochromatin, which is also apparent in Sirt7 KO cells. Relaxation of rDNA heterochromatin causes loss of rDNA repeats and might eventually result in genomic instability, a condition promoting cellular aging [21]. Hence, a pivotal role of Sirt7 for controlling genomic stability emerges, although the mechanisms by which this effect is achieved differ between different chromosomal locations.

Numerous reports described the need for a tight regulation of Sirt1 activity to maintain healthy cellular physiology, which occurs at different levels and by different mechanisms. A recent example is the reciprocal regulation of Sirt1 and the circadian clock protein Per2, which is part of the PER complex involved in histone acetylation and methylation. In the absence of Per2, Sirt1 cannot be efficiently downregulated and vice versa, leading to dysregulation of Sirt1-dependent processes and acceleration of aging [36]. Here, we described a critical role of Sirt7 in the regulation of Sirt1 that plays important roles for the maintenance of cellular homeostasis and for prompt cellular responses to adapt to changing environmental conditions. Such cellular responses require fast regulation of stress-dependent factors, such as sirtuins. We reason that acetylation/deacetylation is a key element of adaptive cellular processes. Suboptimal stress responses might promote gradual accumulation of damage and cause premature aging.

## Materials and methods

### Cell culture and transfection

Primary mouse embryonic fibroblasts (MEFs) were obtained and cultured as described [21]. HEK 293T and U2OS cells were cultured in 4.5 g/L glucose DMEM medium (Sigma-Aldrich) supplemented with 10% fetal calf serum (Sigma-Aldrich), 100U/ml penicillin, 0.1 mg/ml streptomycin and 2 mM glutamine (Sigma-Aldrich) at 37°C in a humidified atmosphere with 5% CO_2_. HEK293T cells were transfected using the calcium phosphate method [37]. U2OS cells were transfected using TurboFect reagent (Thermofisher).

### Plasmids

C-terminal Flag-tagged human Sirt7 was cloned into the pcDNA 3.1+ vector (Invitrogen). C-terminal EGFP-tagged mouse Suv39h1 was cloned into the pcDNA4/to vector (Invitrogen). N-terminal 6x myc-tagged mouse Suv39h1 was cloned into the pCS2+ vector (RZPD). Wild type Flag-Sirt1 construct has been described [19]. The Flag-Sirt1 K230R and Flag- Sirt1 K230Q mutants were obtained from the wild type Flag-Sirt1 using QuickChange site-directed mutagenesis kit (Stratagene). C-terminal 3x HA-tagged mouse Sirt1 was cloned into the pCMV-Sport6 plasmid (Invitrogen).

### Co-immunoprecipitation and Western blotting

Co-immunoprecipitation experiments were performed as described [19,21] in presence of 0.15 U/µL of benzonase (Sigma-Aldrich) using ANTI-FLAG® M2 Affinity Gel (Sigma-Aldrich) followed by elution with FLAG®-peptide (Sigma-Aldrich) or with Anti-Tag (CGY)FP (Evrogen) and anti-Suv39h1 antibody (Novus Biologicals), as indicated. Western blotting was performed as described [19] using the following primary antibodies: ANTI-FLAG® M2 (Sigma-Aldrich), Anti-Tag (CGY)FP (Evrogen), Anti-Sirt7 (Cell Signaling Technology), Anti-Sirt1 (Cell Signaling Technology), Anti-H3K9me3 (Abcam), Anti-RalA (BD Trans. Laboratories; R23520), Anti-Histone 3 (Cell Signaling Technology), anti-HA.11 Tag antibody (Biolegend), anti-Suv39h1 (Cell Signaling Technology), and anti-Suv39h1 (Novus Biologicals). Quantification of the intensity of western blot signals was achieved using Image Lab™ 5.0 software (Bio-Rad).

### Immunofluorescence analysis

Immunofluorescence analysis was performed as previously described [19] using the following primary antibodies: anti-Myc-Tag (Cell Signaling Technology), anti-NPM/B23 (Santa Cruz Biotechnology), anti-H3K9me3 (Abcam), and anti-Heterochromatin Protein-1 α (HP1α) (Millipore).

### Generation of stable cell lines

Stable U2OS cells expressing lentivirus-driven shRNAs were generated as described [21]. The following shRNAs inserted into the pLKO.1 backbone vector (Sigma-Aldrich) were used: scramble shRNA: 5´-CCGGCAACAAGATGAAGAGCACCAACTCGA

GTTGGTGCTCTTCATCTTGTTGTTTTT), human Sirt7 shRNA: 5´-CCGGGTCCAGCCTGAAGGTTCTAAACTCGAGTTTAGAACCTTCAGGC

TGGACTTTTTG-3´. In addition, the following shRNAs inserted into the pGIPZ backbone vector (Dharmacon) were used: scramble shRNA: 5´-TGCTGTTGACAGTGAGCGATCTCG

CTTGGGCGAGAGTAAGTAGTGAAGCCACAGATGTACTTACTCTCGCCCAAGCGAGAGTGCCTACTGCCTCGGA-3´, human Suv39h1 shRNA: 5´- TGCTGTTGACAGTGAGCGACGGGCCTTCGTGTACATCAATTAGTGAAGCCACAGATGTAATTGATGTACACGAAGGCCCGCTGCCTACTGCCTCGGA-3´, human Sirt1 shRNA: 5´- TGCTGTTGA

CAGTGAGCGAGGTGATGAAATTATCACTAATTAGTGAAGCCACAGATGTAATTAGTGATAATTTCATCACCGTGCCTACTGCCTCGGA-3´.

### Chromatin immunoprecipitation (ChIP)

ChIP experiments were performed and analyzed as already described [38] using an anti-H3K9me3 or anti-H3 antibody (Abcam). Immunoprecipitated chromatin was analyzed with primers for human satellite 2 DNA repeats (Sat2 forward: 5′-CATCGAATGGAAATGAAAGGAGTC-3ʹ and Sat2 reverse: 5´- ACCATTGGATGATTGCAGTCAA-3´.

### Statistical analysis

Data are expressed as the mean ± standard deviation of at least 3 independent biological replicates. Statistical significance was assessed by Student’s t-test.
